# Rare variant analysis of blood pressure phenotypes in the Genetic Analysis Workshop 18 whole genome sequencing data using sequence kernel association test

**DOI:** 10.1186/1753-6561-8-S1-S10

**Published:** 2014-06-17

**Authors:** Cates Mallaney, Yun Ju Sung

**Affiliations:** 1 Division of Biostatistics, Washington University in St. Louis, School of Medicine, St. Louis, MO 63110, USA

## Abstract

Sequence kernel association test (SKAT) has become one of the most commonly used nonburden tests for analyzing rare variants. Performance of burden tests depends on the weighting of rare and common variants when collapsing them in a genomic region. Using the systolic and diastolic blood pressure phenotypes of 142 unrelated individuals in the Genetic Analysis Workshop 18 data, we investigated whether performance of SKAT also depends on the weighting scheme. We analyzed the entire sequencing data for all 200 replications using 3 weighting schemes: equal weighting, Madsen-Browning weighting, and SKAT default linear weighting. We considered two options: all single-nucleotide polymorphisms (SNPs) and only low-frequency SNPs. A SKAT default weighting scheme (which heavily downweights common variants) performed better for the genes in which causal SNPs are mostly rare. This SKAT default weighting scheme behaved similarly to other weighting schemes after eliminating all common SNPs. In contrast, the equal weighting scheme performed the best for *MAP4 *and *FLT3*, both of which included a common variant with a large effect. However, SKAT with all 3 weighting schemes performed poorly. Overall power across all causal genes was about 0.05, which was almost identical to the type I error rate. This poor performance is partly due to a small sample size because of the need to analyze only unrelated individuals. Because a half of causal SNPs were not found in the annotation file based on the 1000 Genomes Project, we suspect that performance was also affected by our use of incomplete annotation information.

## Background

Common variants have long been thought to have a major role in common phenotypes; however, genome-wide association studies (GWAS) that focused on common variants have explained only a small proportion of heritability. Because rare variants are expected to have larger effect sizes than common variants, they may explain a proportion of the missing heritability [1]. As reviewed by Bansal *et al *[2], because of limited power for detecting the individual effects of rare variants, several approaches for identifying them have used collapsing methods (called burden tests) that assess the combined effect of multiple rare variants in a genomic region.

Because these burden tests implicitly assume that all variants influence the trait in the same direction and with the same magnitude of effect (after incorporating known weights), their power is reduced if certain variants confer increased risk while others are protective [3]. Several methods have been developed recently that are less sensitive to this mixture of rare-variant effects. Two one-sided statistics by Ionita-Laza *et al *[4] quantify enrichment in risk variants and protective variants, respectively. The C-alpha test by Neale *et al *[5] uses the distribution of genetic variation observed in cases and controls to detect the presence of a mixture, thus implicating genes as risk factors for disease. By testing the variance rather than the mean, the C-alpha test maintains consistent power when the target set contains both risk and protective variants [5]. The sequence kernel association test (SKAT) by Wu *et al *[6] is a score-based variance component test for association of variants in a region for continuous and discrete traits, which easily adjusts for covariates.

Performance of burden tests has been shown to depend on how rare and common variants are weighted when collapsing them in a genomic region [2,7]. In this article, using the Genetic Analysis Workshop 18 (GAW18) sequence data, we investigated whether performance of the most commonly used nonburden test, SKAT, also depends on the weighting scheme. In particular, we evaluated 3 weighting schemes: equal weighting, Madsen-Browning weighting, and SKAT default linear weighting. Analyses were performed without knowledge of the underlying simulation model. However, we used the GAW18 answers in presenting the results below.

## Methods

### Sequence and phenotypic data

GAW18 provided whole genome sequencing (WGS) data and longitudinal phenotype data for related individuals of Mexican American heritage. Because SKAT can only handle unrelated individuals, we used both sequence and phenotype data on a subset of 142 unrelated individuals. Using PLINK [8], we extracted the data and created input files for SKAT. We used the first-visit measurements for diastolic blood pressure (DBP) and systolic blood pressure (SBP) for all 200 simulated data and the most likely genotype data based on sequencing (geno.csv files). For covariates of both DBP and SBP, we used age, sex, blood pressure medication, and smoking.

### Annotation files

To obtain a gene list to run SKAT, we used the annotation file that was constructed based on the 1000 Genomes Project (http://www.sph.umich.edu/csg/abecasis/MACH/download/1000G-2010-06.html). Of the 8,348,674 single-nucleotide polymorphisms (SNPs) in the data set, 2,652,577 were located in genes, with a total of 10,922 genes. Some of these SNPs belonged to multiple overlapping genes and were used for multiple genes in which they were contained. Because 1,856,005 SNPs in the genotype files were not found in the 1000 Genomes annotation file, not all genes were available for testing. For the SBP phenotype, we were missing 4 causal genes, and for the DBP phenotype, we were missing 3 causal genes.

### Statistical analysis

We applied SKAT to the analysis of all 200 replicates of simulated DBP and SBP phenotypes for 2,652,577 SNPs that are located in genes, spanning all odd-numbered chromosomes provided by GAW18. Because our analysis was based on 142 unrelated individuals, instead of using minor allele frequency (MAF) provided by GAW18 (which is based on 959 related individuals), we computed MAF based on these 142 individuals using PLINK [8]. We also constructed a data set including low-frequency SNPs (with our computed MAF <0.05).

**Figure 1:**
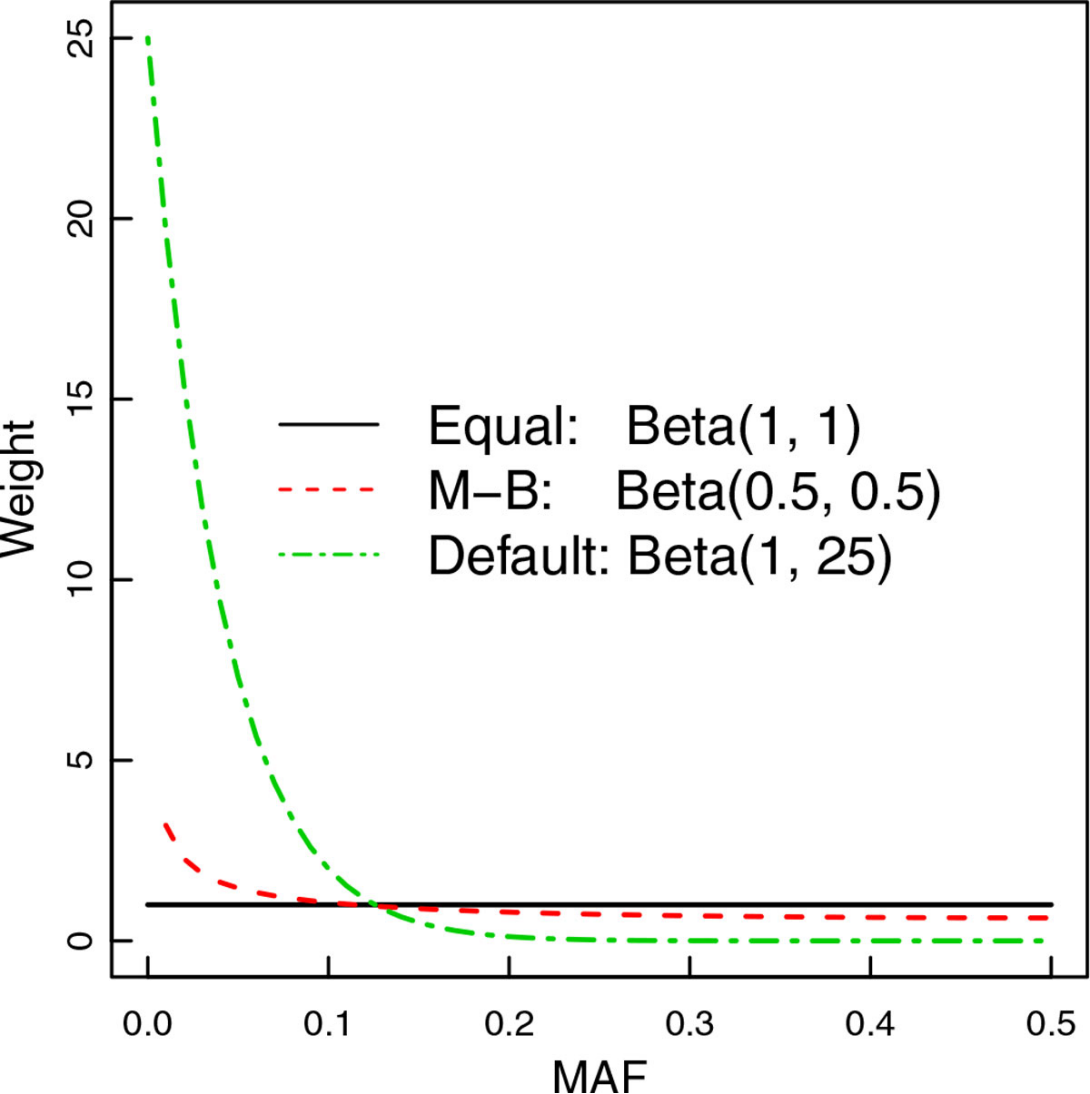
**Weighting schemes used in sequence kernel association test analysis**. *MAF*, minor allele frequency.

For a continuous trait *Y*, SKAT uses a linear model $Y_{i}=\gamma_{0}+\gamma_{1}\;X_{i}+\beta G_{i}$ with genotype values *G_i _*and covariates *X_i _*for subject *i*. As described by Wu *et al *[6], SKAT assumes that the genetic effect *β_j _*of an individual variant *j *follows an arbitrary distribution with mean 0 and variance *w_j_τ*, where *τ *is a variance component and *w_j _*is a prespecified weight for variant *j*. SKAT further assumes that $\sqrt{w_{j}}$ follows *Beta (MAF_j_; a_1_,a_2_)*. Weighting with *a_1 _= a_2 _= *1 corresponds to equally weighting all variants regardless of their MAF, which was shown to be equivalent to the C-alpha test by Neale *at al *[5]. Weighting with *a_1 _= a_2 _= *0.5 is the same as the weight used by Madsen and Browning [7]. Default linear weighting by SKAT uses *a_1 _*= 1 and *a_2 _*= 25, which puts more weight on rare variants than Madsen-Browning (M-B) weighting, as shown in Figure 1.

For both data sets (one with all SNPs and another with only low-frequency SNPs), we ran SKAT using these 3 weighting schemes: equal weighting, Madsen-Browning weighting, and SKAT default linear weighting. For each scenario, we obtained *p*-values for the 10,922 genes in 200 replications. To evaluate performance, we computed power (true-positive) and type I error (false-positive) rates at level 0.05. For each gene, power was computed by the proportion of replicates with *p *< 0.05 over 200 replicates. The overall power was computed by averaging these values across all causal genes. Type I error was computed by averaging these values across all null genes.

## Results

### Overall empirical power and type I error

Table 1 presents type I error and overall power using 3 weighting schemes for both data sets. All 3 weighting schemes performed poorly. No causal gene met the threshold for genome-wide significance, and the lowest *p*-value for a true positive occurred at 1.0 × 10^−5 ^for analyses of SBP and 1.0 × 10^−4 ^for analyses of DBP. The type I error was slightly below 0.05, roughly keeping the level 0.05. Overall power across all causal genes was about 0.05, which was almost identical to the type I error rate. The equal weighting scheme using all SNPs performed slightly better for both DBP and SBP phenotypes.

**Table 1:** Empirical power and type I error at α = 0.05 for diastolic blood pressure and systolic blood pressure phenotypes

Phenotype	DBP						SBP					
**SNPs**	**All**			**Rare**			**All**			**Rare**		

**Weight**	**Equal**	**M-B**	**Default**	**Equal**	**M-B**	**Default**	**Equal**	**M-B**	**Default**	**Equal**	**M-B**	**Default**

Type I Error	0.047	0.043	0.043	0.044	0.042	0.043	0.048	0.048	0.049	0.048	0.050	0.049
Power	**0.053**	0.050	0.048	0.048	0.050	0.048	**0.054**	0.053	0.053	0.049	0.052	0.052

Bolded text indicates the highest power across 6 analysis options for each phenotype.

*DBP*, diastolic blood pressure; *M-B*, Madsen-Browning; *SBP*, systolic blood pressure; *SNP*, single-nucleotide polymorphism.

### Empirical power at causal genes

**Figure 2:**
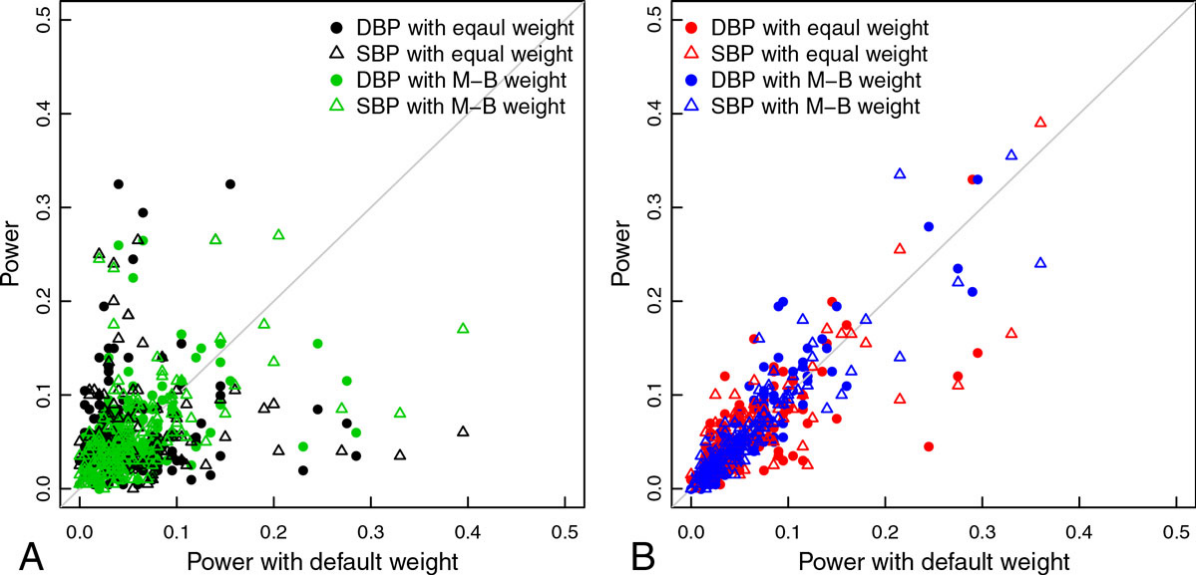
**Power comparison using 3 weighting schemes**. (A) All single-nucleotide polymorphisms (SNPs). (B) Rare SNPs. *DBP*, diastolic blood pressure *M-B*, Madsen-Browning; *SBP*, systolic blood pressure.

Although overall power was similar across the 3 weighting schemes, power at each causal gene varied greatly and depended on the weighting scheme. When using all SNPs, results were considerably different across 3 weighting schemes, as shown in the left plot of Figure 2. In particular, the correlation between power using equal weighting and power using default weighting was 0.17 (shown in Table 2). In contrast, when using rare SNPs, results were more consistent across different weighting schemes, as shown in the right plot of Figure 2. Results using default weighting were very similar when using all SNPs and when using only rare SNPs (with correlation 0.97).

**Table 2:** Spearman correlation across empirical powers at all causal genes

Correlation		All			Rare		
		**Equal**	**M-B**	**Default**	**Equal**	**M-B**	**Default**

All	Equal		0.69	0.17	0.13	0.11	0.11
	M-B			0.61	0.47	0.55	0.54
	Default				0.79	**0.87**	**0.95**

Rare	Equal					0.70	**0.80**
	M-B						**0.91**
	Default						

Bolded text indicates the correlation greater than 0.8.

*M-B*, Madsen-Browning

To understand how power depended on the weighting scheme, we selected causal genes that show power over 0.2 from at least one of weighting schemes from Figure 2; these selected causal genes are presented in Table 3. For genes *TXNIP, GLUL, PDCD6IP, DGKE*, and *SCAP*, the SKAT default weighting gave much higher power than equal weighting. Except for *PDCD6IP*, all causal SNPs were low frequency (with MAF <0.05). Using only low-frequency SNPs provided similar performance.

**Table 3:** Causal genes with empirical power over 0.2 using any weighting scheme

Causal Genes	Number of SNPs		Number of causal SNPs^1^		Total % variance explained^2^	All			Rare		
					
	All	Rare	All	Rare		Equal	M-B	Default	Equal	M-B	Default
**DBP**											
*MAP4*	542	392	6	5	0.04826	**0.30**	**0.27**	0.07	0.09	0.06	0.07
*FLT3*	639	308	4	2	0.01053	**0.33**	**0.26**	0.04	0.06	0.04	0.04
*DHX8*	142	89	2	2	0.00007	**0.25**	**0.23**	0.06	0.09	0.04	0.05
*FGR*	92	78	2	1	0.00006	**0.33**	0.12	0.16	0.18	0.11	0.16
*TXNIP*	9	6	1	1	<1.0E-5	0.09	0.16	**0.25**	**0.33**	**0.21**	**0.29**
*GLUL*	44	24	2	2	0.00012	0.07	0.12	**0.28**	0.12	**0.24**	**0.28**
*PDCD6IP*	359	215	4	2	0.00058	0.04	0.06	**0.29**	0.15	**0.33**	**0.30**
*DGKE*	136	71	1	0	0.00005	0.02	0.05	**0.23**	0.05	**0.28**	**0.25**
*PTTG1IP*	174	116	2	2	0.00005	0.04	0.09	0.15	**0.20**	0.13	0.15
*EPHA2*	133	93	1	1	0.00021	0.03	0.17	0.11	0.03	**0.20**	0.10

**SBP**											
*MAP4*	542	392	7	5	0.05824	**0.27**	**0.27**	0.14	0.17	0.09	0.14
*FLT3*	639	308	4	2	0.00841	**0.20**	0.18	0.04	0.04	0.03	0.03
*DHX8*	142	89	2	2	0.00002	**0.25**	**0.25**	0.02	0.10	0.03	0.03
*S100A6*	14	11	2	2	0.00003	**0.27**	0.09	0.06	0.05	0.05	0.05
*KRT23*	79	31	2	1	0.00016	**0.24**	**0.24**	0.04	0.03	0.02	0.02
*TXNIP*	9	6	1	1	<1.0E-5	0.09	0.14	**0.20**	**0.26**	0.14	**0.22**
*GLUL*	44	24	2	2	0.00006	0.04	0.09	**0.27**	0.11	**0.22**	**0.28**
*PDCD6IP*	359	215	4	2	0.00027	0.04	0.08	**0.33**	0.17	**0.36**	**0.33**
*SCAP*	114	83	1	1	0.00003	0.06	0.17	**0.40**	**0.39**	**0.24**	**0.36**
*EPHA2*	133	93	1	1	0.00018	0.04	**0.27**	**0.21**	0.10	**0.34**	**0.22**

^1^Number of causal single-nucleotide polymorphisms (SNPs) is computed based on the used annotation file (not based on the answers).

^2^Total % variance explained is computed by adding the percent variance explained by each SNP based on the used annotation file (not based on the answers).

Bolded text indicates the empirical power greater than 0.2.

*DBP*, diastolic blood pressure; *M-B*, Madsen-Browning; *SBP*, systolic blood pressure.

However, for genes *MAP4, FLNB, DHX8, FGR, S100A6*, and *KRT23*, the equal weighting scheme gave much higher power than the SKAT default weighting (0.32 vs. 0.04 at *MAP4 *for DBP). Looking within the top 20 causal variants, we found that *MAP4 *has 6 of the top 10 variants with the greatest effect on SBP and DBP. For all of these variants, one was common with a MAF of 0.38, and the other 5 were low frequency (with MAF <0.05). Similarly, gene *FLT3 *had 2 variants that fell within the top 20 causal variants, one common with a MAF of 0.42 and one rare with a MAF of 0.0016. No other causal gene had more than one variant in the top 20 causal variants. Because of this, we suspect that equal weighting performed better than SKAT default weighting, which gives a much higher weight to rarer variants.

We also considered whether performance would depend on the signal-to-noise ratio, defined as the number of causal variants divided by the total number of variants in the gene, for each of the top 15 genes. For DBP, *MAP4 *had the 10th lowest sig signal-to-noise ratio, and *FLT3 *had the 13th lowest. For the SBP phenotype, they were 7th and 10^th^, respectively. Therefore, we found that signal-to-noise ratio could not account for the relatively high performance on *MAP4 *and *FLT3*.

## Discussion and conclusions

SKAT has become one of the most commonly used nonburden tests for analyzing rare variants because it is fast and because nonburden tests are shown to be more powerful when most variants are noncausal or the effects of causal variants are in different directions. Using SKAT, we were able to analyze the entire sequencing data for all 200 replications of both DBP and SBP simulated phenotypes under six different cases (3 weighting schemes times two data sets: one with all SNPs another with only low-frequency SNPs). Performance of burden tests has been shown to depend on how to weigh rare and common variants when collapsing them in a genomic region. In this article, using the GAW 18 sequence data, we found that performance of the nonburden test SKAT also depended on the weighting scheme.

In this article, we focused on the ability of SKAT to detect genes with various MAF-based weighting schemes. When causal SNPs within a gene are mostly rare, then MAF-based weighting by upweighting rare variants and downweighting common variants would be more desirable. Indeed we found that the SKAT default weighting scheme (which heavily downweights common variants) performed better for such genes. We also found that this SKAT default weighting scheme behaved similarly to other weighting schemes after eliminating all common SNPs. In contrast, we observed that an equal weighting scheme performed the best for *MAP4 *and *FLT3*, both of which included a common variant with a large effect.

However, we found that SKAT with all 3 weighting schemes performed poorly. Overall power across all causal genes was about 0.05, which was almost identical to the type I error rate. We suspect that this poor performance is partly due to a small sample size. Wu *et al *[6] previously demonstrated that SKAT, as with other rare variant methods, is severely hindered by small sample sizes, and this data set was no exception. Lin and Tang [9] had previously compared several rare variant methods and found that SKAT is very conservative when sample size and the level α for calculating power are small. Our results were also consistent with a study performed by Daye *et al *[10] that showed at a sample size of 200, SKAT performs at or around the α = 0.05 level regardless of coverage.

Many GAW18 investigators that analyzed only unrelated individuals (with a sample size of 142) have also observed poor performance. Because of high sequencing costs, small sample sizes will continue to be a problem with rare variant analysis. We observed that analysis using all related individuals (with a sample size of 847) performed much better [11]. For example, results based on a burden test using all related individuals provided an empirical power of 0.99 for *MAP4*. SKAT was recently extended to handle related individuals for continuous phenotypes [12]. We expect that if the extended SKAT were applied to all related individuals in GAW18 data, it would provide better performance for detecting rare variants.

To apply both burden and nonburden tests for a gene, it is necessary to know which SNPs are contained in the gene. GAW18 performed WGS of 1043 individuals of Mexican American heritage with an average 60x sequencing depth, with a goal of finding novel SNPs. However, this creates a problem for applying these rare variant approaches because available annotation information does not contain these novel variants. In particular, among 1458 causal SNPs in the Answers, only 731 SNPs were contained in the annotation file that was constructed based on the 1000 Genomes Project. In addition to a small sample size, we suspect that our results were also affected by our use of incomplete annotation information. Although the results and issues presented in this article were based on GAW18 data, they may be shared with other sequencing studies.

## Competing interests

The authors declare that they have no competing interests.

## Authors' contributions

CM performed the statistical analyses and drafted the manuscript. YJS designed the overall study and drafted the manuscript. Both authors read and approved the final manuscript.

## References

[B1] Manolio TA Collins FS Cox NJ Goldstein DB Hindorff LA Hunter DJ McCarthy MI Ramos EM Cardon LR Chakravarti A Finding the missing heritability of complex diseases Nature 2009 461 747 753 10.1038/nature08494 19812666 PMC2831613

[B2] Bansal V Libiger O Torkamani A Schork NJ Statistical analysis strategies for association studies involving rare variants Nat Genet 2010 11 773 785 10.1038/nrg2867 PMC3743540 20940738

[B3] Cohen JC Boerwinkle E Mosley TH Hobbs HH Sequence variations in PCSK9, low LDL, and protection against coronary heart disease N Engl J Med 2006 354 1264 1272 10.1056/NEJMoa054013 16554528

[B4] Ionita-Laza I Buxbaum JD Laird NM Lange C A new testing strategy to identify rare variants with either risk or protective effect on disease PLoS Genet 2011 7 e1001289 10.1371/journal.pgen.1001289 21304886 PMC3033379

[B5] Neale BM Rivas MA Voight BF Altshuler D Devlin B Testing for an unusual distribution of rare variants PLoS Genet 2011 7 e1001322 10.1371/journal.pgen.1001322 21408211 PMC3048375

[B6] Wu MC Lee S Cai T Li Y Boehnke M Lin X Rare variant association testing for sequencing data using the sequence kernel association test (SKAT) Am J Hum Genet 2011 89 82 93 10.1016/j.ajhg.2011.05.029 21737059 PMC3135811

[B7] Madsen BE Browning SR A groupwise association test for rare mutations using a weighted sum statistic PLoS Genet 2009 5 e1000384 10.1371/journal.pgen.1000384 19214210 PMC2633048

[B8] Purcell S Neale B Todd-Brown K Thomas L Ferreira MAR Bender D Maller J Sklar P de Bakker PIW Daly MJ PLINK: a toolset for whole-genome association and population-based linkage analysis Am J Hum Genet 2007 81 559 575 10.1086/519795 17701901 PMC1950838

[B9] Lin DY Tang ZZ A general framework for detecting disease associations with rare variants in sequencing studies Am J Hum Genet 2011 89 354 367 10.1016/j.ajhg.2011.07.015 21885029 PMC3169821

[B10] Daye ZJ Li H Wei Z A powerful test for multiple rare variants association studies that incorporates sequencing qualities Nucleic Acids Res 2012 40 e60 10.1093/nar/gks024 22262732 PMC3340416

[B11] Sung YJ Basson J Rao DC Whole genome sequence analysis of the simulated systolic blood pressure in Genetic Analysis Workshop 18 family data: long-term average and collapsing methods BMC Proc 2014 8 Suppl 1 S12 10.1186/1753-6561-8-S1-S12 PMC4143632 25519365

[B12] Chen H Meigs JB Dupuis J Sequence kernel association test for quantitative traits in family samples Genet Epidemiol 2013 37 196 204 10.1002/gepi.21703 23280576 PMC3642218

